# Lipid biomarkers that reflect postoperative recurrence risk in lung cancer patients who smoke: a case–control study

**DOI:** 10.1186/s12944-023-01778-3

**Published:** 2023-01-28

**Authors:** Yusuke Takanashi, Tomoaki Kahyo, Takamitsu Hayakawa, Keigo Sekihara, Akikazu Kawase, Minako Kondo, Takuya Kitamoto, Yutaka Takahashi, Tomohito Sato, Haruhiko Sugimura, Norihiko Shiiya, Mitsutoshi Setou, Kazuhito Funai

**Affiliations:** 1 First Department of Surgery, Hamamatsu University School of Medicine, 1-20-1 Handayama, Higashi Ward, Hamamatsu, Shizuoka 431-3192 Japan; 2 Department of Cellular and Molecular Anatomy, Hamamatsu University School of Medicine, 1-20-1 Handayama, Higashi Ward, Hamamatsu, Shizuoka 431-3192 Japan; 3 International Mass Imaging Center, Hamamatsu University School of Medicine, 1-20-1 Handayama, Higashi Ward, Hamamatsu, Shizuoka 431-3192 Japan; 4 Advanced Research Facilities & Services, Hamamatsu University School of Medicine, 1-20-1 Handayama, Higashi Ward, Hamamatsu, Shizuoka 431-3192 Japan; 5 Department of Tumor Pathology, Hamamatsu University School of Medicine, 1-20-1 Handayama, Higashi Ward, Hamamatsu, Shizuoka 431-3192 Japan; 6 Department of Systems Molecular Anatomy, Institute for Medical Photonics Research, Hamamatsu University School of Medicine, 1-20-1 Handayama, Higashi Ward, Hamamatsu, Shizuoka 431-3192 Japan

**Keywords:** Lung cancer, Cigarette smoking, Recurrence risk, Lipid biomarker, Mass spectrometry

## Abstract

**Background:**

The risk of postoperative recurrence is higher in lung cancer patients who smoke than non-smokers. However, objective evaluation of the postoperative recurrence risk is difficult using conventional pathological prognostic factors because of their lack of reproducibility. Consequently, novel objective biomarkers that reflect postoperative risk in lung cancer patients who smoke must be identified. Because cigarette smoking and oncogenesis alter lipid metabolism in lung tissue, we hypothesized that the lipid profiles in lung cancer tissues are influenced by cigarette smoking and can reflect the postoperative recurrence risk in smoking lung cancer patients. This study aimed to identify lipid biomarkers that reflect the smoking status and the postoperative recurrence risk.

**Methods:**

Primary tumor tissues of lung adenocarcinoma (ADC) (*n* = 26) and squamous cell carcinoma (SQCC) (*n* = 18) obtained from surgery were assigned to subgroups according to the patient’s smoking status. The ADC cohort was divided into never smoker and smoker groups, while the SQCC cohort was divided into moderate smoker and heavy smoker groups. Extracted lipids from the tumor tissues were subjected to liquid chromatography-tandem mass spectrometry analysis. Lipids that were influenced by smoking status and reflected postoperative recurrence and pathological prognostic factors were screened.

**Results:**

Two and 12 lipid peaks in the ADC and SQCC cohorts showed a significant positive correlation with the Brinkman index, respectively. Among them, in the ADC cohort, a higher lipid level consisted of three phosphatidylcholine (PC) isomers, PC (14:0_18:2), PC (16:1_16:1), and PC (16:0_16:2), was associated with a shorter recurrence free period (RFP) and a greater likelihoods of progressed T-factor (≥ pT2) and pleural invasion. In the SQCC cohort, a lower *m/z* 736.5276 level was associated with shorter RFP and greater likelihood of recurrence.

**Conclusions:**

From our data, we propose three PC isomers, PC (14:0_18:2), PC (16:1_16:1), and PC (16:0_16:2), and a lipid peak of *m/z* 736.5276 as novel candidate biomarkers for postoperative recurrence risk in lung ADC and SQCC patients who are smokers.

**Supplementary Information:**

The online version contains supplementary material available at 10.1186/s12944-023-01778-3.

## Background

Cigarette smoking is a strong risk factor for developing non-small cell lung cancer (NSCLC). Around 90% of NSCLC cases are associated with active cigarette smoking [1]. Although the standard treatment modality for resectable stage I–III NSCLC is radical surgery [2], the postoperative recurrence risk for current smokers is reported to be high compared with non-smokers and former smokers (relative risks of recurrence: current smoker, 1; non-smoker, 0.45; former smoker; 0.54) [3]. Hence, predicting the postoperative recurrence risk in individual NSCLC patients who are smokers will contribute to developing a qualified postoperative therapeutic strategy.

Currently, the postoperative recurrence risk of NSCLC patients is evaluated using the tumor, lymph node, and metastasis (TNM) classification system [4] and several histopathological prognostic factors, such as lymph node metastasis [5], pleural invasion [6], lymphatic vessel invasion [7], blood vessel invasion [8, 9], and spread through air space (STAS) [10]. However, evaluating the risk of postoperative recurrence using these conventional prognostic factors remains challenging because of subjective judgment and a lack of reproducibility [11]. Therefore, identifying novel and objective biomarkers that reflect postoperative recurrence risk in smokers NSCLC patients is needed.

With the recent advancements in mass spectrometry technology, lipidomics has been utilized as a new field for exploring cancer biomarkers [12]. Cancer cells exhibit a common phenotype of uncontrolled proliferation, and their lipid metabolism is altered to generate energy and biomass components efficiently [13, 14]. Specific lipids in cancer tissues have been reported as potential biomarkers reflecting patient prognosis and the risk of postoperative recurrence. For example, an elevated level of phosphatidylcholine (PC) (32:1) is suggested to be a candidate predictor for postoperative recurrence of primary triple-negative breast cancer [15]. In clear cell renal cell carcinoma, oleic acid attenuation is associated with a shorter progression-free period [16]. In the field of NSCLC, lung cancer tissue has been demonstrated to have distinct lipid profiles for their cancer differentiation [17–20], histological subtypes [17–20], and oncogenic driver gene mutation [20], reflecting their lipid metabolism alterations. Furthermore, we have reported specific sphingomyelin (SM) species in lung adenocarcinoma (ADC) and squamous cell carcinoma (SQCC) as novel candidate predictors for postoperative recurrence after radical surgery [21, 22]. Thus, these previous works led us to focus on lipidomics to explore novel biomarkers that reflect postoperative recurrence risk in smoking NSCLC patients.

Cigarette smoking alters lipid metabolism in the lung tissue. Short-term cigarette exposure induces decreased PC levels in the alveolar type II cells of the mouse lung [23, 24]. Additionally, decreased levels of surfactant PC are observed in bronchoalveolar lavage fluids from human lungs exposed to cigarette smoke [25]. PC is a significant surfactant component [23–25], and specific PC species can suppress lung cancer growth and metastasis by decreasing antiapoptotic factors and matrix metallopeptidases [26, 27]. From these previous studies, we hypothesized that NSCLC tissue lipid profiles are influenced by cigarette smoking and reflect the risk of postoperative recurrence in NSCLC smoking patients. However, the influence of cigarette smoking on the lipid profiles of lung cancer tissues has not been previously investigated.

Therefore, this study aims to identify NSCLC lipid biomarkers that can reflect the patient’s smoking status and postoperative recurrence risk using liquid chromatography-tandem mass spectrometry (LC–MS/MS). Such biomarkers may contribute to evaluating postoperative recurrence risk and developing a qualified postoperative therapeutic strategy for smoking NSCLC patients.

## Methods

### Patients and tissue samples

Among lung ADC and SQCC patients who received surgery with complete resection from January 2013 to December 2016 at Hamamatsu University Hospital, cases with available retrospective frozen tissue samples were enrolled. Primary tumor tissue samples were frozen in liquid nitrogen immediately after resection and stored at -80 °C. Postoperative patients were followed-up with computed tomography (CT) and blood examination of carcinoembryonic antigen (CEA), squamous cell carcinoma antigen (SCC), and cytokeratin 19 fragment (CYFRA) every three months during the first two years, then every six months until five years after the surgery. Tumor marker elevation (CEA ≥ 5.0 ng/mL, SCC ≥ 2.5 ng/mL, CYFRA ≥ 3.5 ng/mL) without recurrent findings on CT was screened for brain or bone metastasis with head magnetic resonance imaging (MRI) and systemic positron emission tomography (PET).

For patient selection, we retrospectively enrolled frozen tissue samples obtained from NSCLC patients with ADC or SQCC. Patients who received induction chemotherapy or radiotherapy, which could cause artifacts in lipid analysis, were excluded. We defined recurrence as radiological imaging-based findings of distant or locoregional recurrence within five years after surgery.

### Histopathological evaluation

Three μm-thick sections of paraffin-embedded tissue blocks were used. Hematoxylin and eosin (H&E) staining was used to evaluate the adenocarcinoma subtype, tumor size, lymph node metastasis, and STAS. D2-40 and Elastica van Gienson staining were used to assess lymphatic and blood vessel invasion, respectively. For the enrolled cases, histopathological diagnoses and pathological staging were performed by experienced pathologists according to the World Health Organization criteria [28] and the 8th edition of the TNM classification for lung and pleural tumors [4], respectively. In principle, an adenocarcinoma phenotype was confirmed by the positivity of thyroid transcription factor-1 (TTF-1) or napsin A. On the other hand, a squamous phenotype was confirmed with the positivity of one of the squamous markers (p40, p63, and cytokeratin 5/6) and negativity for TTF-1.

### Lipid extraction from frozen tissue samples

As described previously, lipids were extracted from the frozen tissue samples [21, 22]. Briefly, each tissue was weighed using a Sartorius analytical lab balance CPA224S (Sartorius AG, Göttingen, Germany) (Additional file 1, Supplemental Fig. 1). Then, the modified Bligh-Dyer method was applied to the tissue samples for lipid extraction. During the extraction, 1.6 mmol of 1,2-dilauroyl-sn-glycero-3-PC (Avanti Polar Lipids, Alabaster, AL, USA), PC (12:0_12:0) per 1 mg of sample was added to standardize the lipid levels. The extracted lipids were dried using miVac Duo LV (Genevac, Ipswich, UK). We dissolved the dried lipids with methanol proportionally to the tissue weights to keep the PC (12:0_12:0) levels similar among the cases.

### Lipid analysis by LC–MS/MS

As reported previously, the diluted lipids were subjected to LC–MS/MS [21, 22]. Briefly, 2–10 μL of the diluted lipids were applied to an Acclaim 120 C18 column (150 mm × 2.1 mm, three μm) (Thermo Fisher Scientific, Waltham, MA, USA) and analyzed using a Q Exactive™ Hybrid Quadrupole-Orbitrap™ Mass Spectrometer (Thermo Fisher Scientific) with an UltiMate 3000 (Thermo Fisher Scientific). Spectral data were recorded using Xcalibur v3.0 Software (Thermo Fisher Scientific). Lipid peak identification and semi-quantification were performed using LipidSearch™ software version 4.2.13 (Mitsui Knowledge Industry, Tokyo, Japan) with the same parameter settings as reported previously [21, 22]. We aligned the identified peaks with a retention time (RT) tolerance of 1.0 min. Identified redundant peaks with different RTs were regarded as independent structural isomers (shown as “Duplication” in Additional File 2, sheet 1). The relative abundance of the identified lipid peaks was measured by calculating their area value. An area value of a lipid species was divided by that of the standard PC (12:0_12:0) in the corresponding case for standardization. The complete list of identified lipid peaks with standardized area values is presented in Additional File 2, sheet 1.

### Data processing

To screen lipids influenced by cigarette smoking, ADC and SQCC patient cohorts were divided into two subgroups according to the smoking status. The ADC cohort was divided into never smoker (Brinkman index [BI] = 0) and smoker (BI > 0) groups to detect the influence of cigarette smoking on lipid profiles as much as possible. On the other hand, the SQCC cohort was divided into moderate smoker (BI < 1590) and heavy smoker (BI > 1590) groups bordering on a median BI value of 1590, as the cohort did not include never smokers. *P*-values were calculated using Welch’s t-test between the subgroups for each area value of lipid species. Additionally, the fold change of respective lipid peaks was calculated as follows: mean area value of smoker divided by that of never smoker in the ADC cohort; mean area value of heavy smoker divided by that of moderate smoker in the SQCC cohort. Then, volcano plots with -log10 (*P*-value) for the vertical axis and log2 (fold change) for the horizontal axis were generated for the two cohorts, and significantly abundant lipids in the respective subgroups were screened. *P*-values of < 0.05 and fold changes ≥ 2.0 or ≤ 0.5 were considered significant. The heat map display and Pearson correlation analysis of the relative intensities of the screened significant lipid species and BI were performed using MetaboAnalyst 5.0 (https://www.metaboanalyst.ca/). The lipid peak intensity values were divided by each lipid peak's standard deviation for standardization. Thus, lipid peaks significantly correlated with BI (Pearson correlation coefficient ≥ 0.5 ∩ *P*-value < 0.05) were selected as lipids influenced by cigarette smoking.

We evaluated the postoperative recurrence risk by examining endpoints, including recurrence-free period (RFP) and odds ratios (ORs) on recurrence, pathological T-factor, lymph node metastasis, pleural invasion, blood vessel invasion, lymphatic vessel invasion and STAS. To calculate the odds ratio (OR), all pathological prognostic factors as objective variables were binarized as follows: T-factor; ≤ pT1 and ≥ pT2, differentiation; ≤ G1 and ≥ G2, and all other factors into negative and positive.

### Statistical analysis

Associations between patient characteristics and smoking status were evaluated by the Fisher exact test (categorical variables) or Mann–Whitney U-test (continuous variables). The Welch’s t-test used in the volcano plots was performed with “TTEST” of Excel™ (Microsoft, Redmond, WA, USA). The cutoff values discriminating the recurrent and non-recurrent cases were calculated by receiver operating characteristic (ROC) curve analysis. The area under the ROC curve (AUC) values were calculated for the discrimination ability. The RFP was determined as the time from operation until the first disease recurrence. RFP curves were generated using the Kaplan–Meier (KM) method. Correlation analyses were performed by calculating Spearman’s rank correlation coefficient (rs). Except for Welch’s t-test, all statistical analyses were performed using R (The R Foundation for Statistical Computing, Vienna, Austria, version 3.6.2). *P*-values < 0.05 were considered statistically significant.

## Results

### Patient clinicopathological characteristics

Patient characteristics are shown in Table 1. Overall, 26 patients in the ADC cohort were divided into never smoker (*n* = 8) and smoker (*n* = 18) groups, while 18 patients in the SQCC cohort were divided into moderate smoker (*n* = 9) and heavy smoker (*n* = 9) groups. In the ADC cohort, more female patients were enrolled in the never smoker group (*n* = 6 [75%]) than in the smoker group (*n* = 1 [5.9%]). Median BI values were 0 (never smoker) and 663 (smoker) in the ADC cohort and 920 (moderate smoker) and 2100 (heavy smoker) in the SQCC cohort. No significant difference was observed in any other characteristic examined. The median follow-up period for the entire cohort was 60 months (range of 4–90 months).

**Table 1:** Patient characteristics of the ADC and SQCC cohorts

Characteristics	ADC (*n* = 26)			SQCC (*n* = 18)		
	**non-smoker (*n* = 8)**	**smoker (*n* = 18)**	***P*-value**	**moderate smoker (*n* = 9)**	**heavy smoker (*n* = 9)**	***P*-value**
Median age (range)	69 (66–80)	71 (48–89)	0.597	71 (54–83)	71 (59–83)	0.626
Sex (male/female)	2/6	17/1	< 0.001	8/1	9/0	1.000
Median Brinkman index (range)	0	663 (40–1600)	< 0.001	920 (180–1530)	2100 (1650–3000)	< 0.001
Pathological stage			0.130			0.128
IA	5	8		4	5	
IB	0	6		1	4	
IIA	0	0		1	0	
IIB	3	2		3	0	
IIIA	0	2		0	0	
Lymph node metastasis (N0/N1/N2)	5/3/0	15/2/1	0.380	6/3/0	9/0/0	0.206
Degree of differentiation (G1/G2/G3)	4/3/1	2/11/5	0.130	2/5/2	2/6/1	1.000
Pleural invasion (pl0/pl1/pl2/pl3)	6/2/0/0	9/7/1/1	0.839	6/2/1/0	5/4/0/0	0.620
Lymphatic vessel invasion (+/-)	3/5	10/8	1.000	4/5	4/5	1.000
Blood Vessel invasion (+/-)	4/4	8/10	1.000	6/3	8/1	0.576
Spread through air space (+/-)	1/7	11/7	0.360	1/8	1/8	1.000
Histologic subtype of ADC			0.205			-
Lepidic	3	1		-	-	
Papillary	4	10		-	-	
Acinar	1	4		-	-	
Solid	0	3		-	-	
Adjuvant chemotherapy						
Indication	7	15	1.000	6	6	1.000
Received	4	6	0.664	1	0	1.000
Recurrent (+/-)	4/4	7/11	0.683	2/7	3/6	1.000
Locoregional alone	1	2	1.000	0	1	1.000
Distant metastasis alone	3	4	0.635	1	1	1.000
Locoregional + distant metastasis	0	1	1.000	1	1	1.000

*Abbreviations*: *ADC* Adenocarcinoma, *SQCC* Squamous cell carcinoma

### Screening of lipids influenced by cigarette smoking

**Fig. 1:**
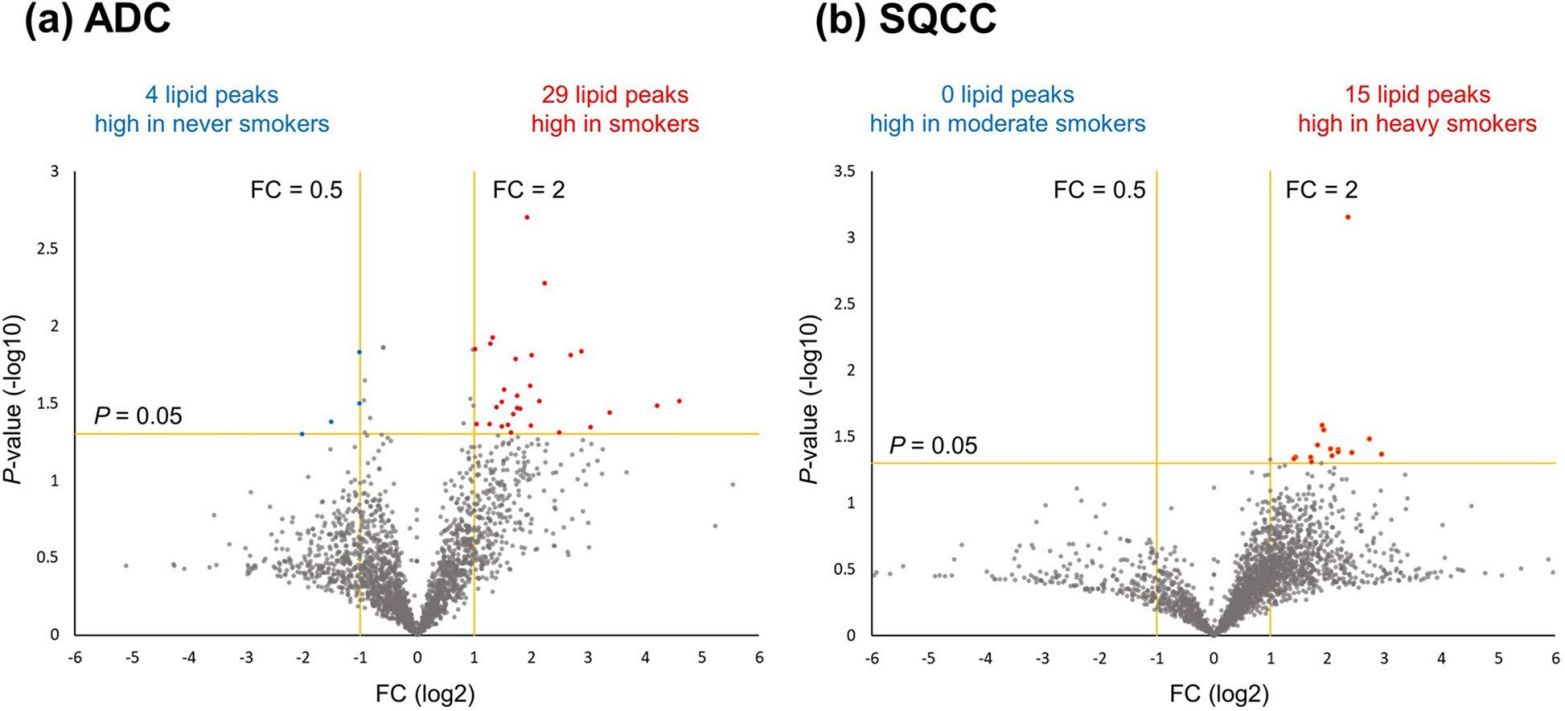
Volcano plots comparing the intensities of the 2,453 identified lipid peaks between the never smoker and smoker groups in the ADC cohort, and between the moderate smoker and heavy smoker groups in the SQCC cohort. **a** In the ADC cohort, four (blue symbols) and 29 (red symbols) lipid peaks were significantly high in the never smoker and smoker groups. **b** In the SQCC cohort, 15 lipid peaks were significantly high in the heavy smoker group, while no significant lipid peaks were identified in the moderate smoker group. The significance was determined as *P*-values of < 0.05, FC of ≥ 2.0 or ≤ 0.5. Abbreviations: ADC, Adenocarcinoma; FC, Fold change; SQCC, Squamous cell carcinoma

**Fig. 2:**
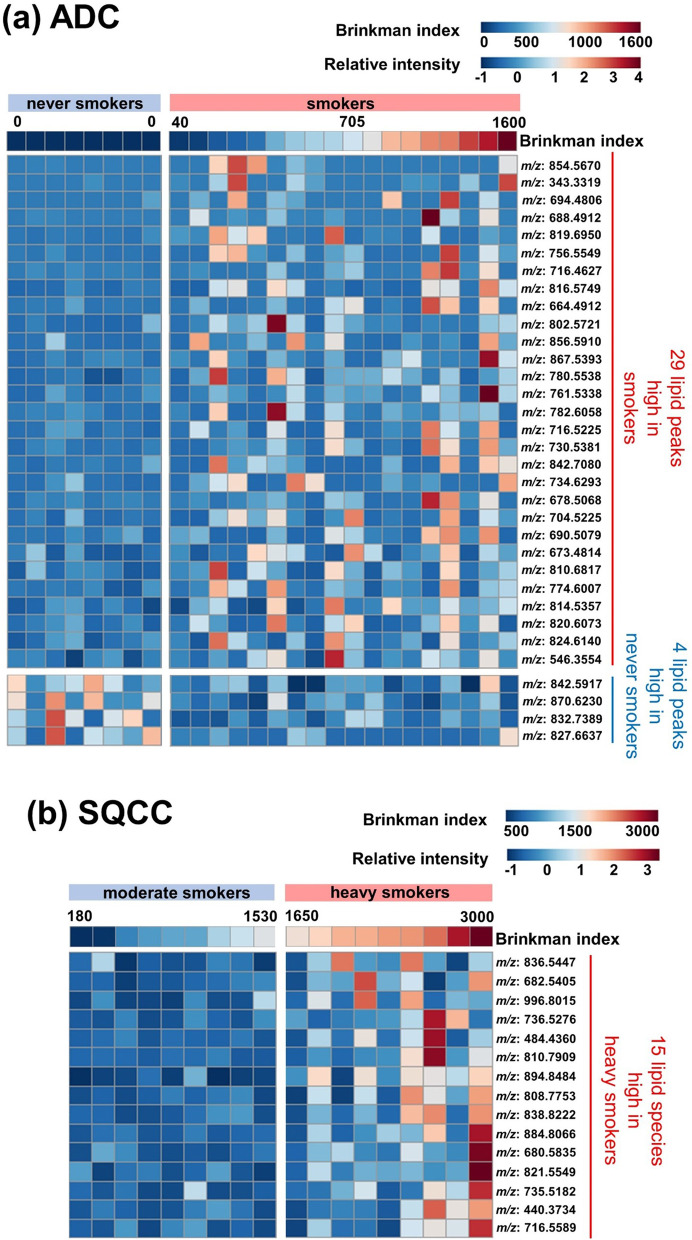
Heat map showing the relative intensities of the screened lipid peaks and Brinkman index (BI) values. The majority of high lipid peaks in the heavy smoker group of the SQCC cohort had apparent positive correlation trends with BI (**b**). In contrast, a weak positive and negative correlation trend was observed in the smoker group of the ADC cohort (**a**). Abbreviations: ADC, Adenocarcinoma; BI, Brinkman index; *m/z*, Mass-to-charge ratio; SQCC, Squamous cell carcinom

In the LC–MS/MS analysis of the lipids extracted from the frozen tissue samples, LipidSearch™ software identified a total of 2,453 lipid peaks. The complete list of the identified lipid peaks is presented in Additional file 2, sheet 1. Lipid intensities between the never smoker and smoker groups in the ADC cohort and between the moderate smoker and heavy smoker groups in the SQCC cohort were compared using volcano plots (Fig. 1). In the ADC cohort, four and 29 significantly high lipid peaks were identified in the never smoker and smoker groups, respectively. In the SQCC cohort, 15 lipid peaks were significantly high in the heavy smoker group, but no significant lipid peaks were identified in the moderate smoker group. The relative intensities of these screened lipid peaks and BI values are depicted using heat maps (Fig. 2). Overall, each screened lipid peak reproducibly showed different intensities between the subgroups in the ADC (Fig. 2a) and SQCC (Fig. 2b). Intriguingly, the majority of high-intensity lipid peaks in the heavy smoker group of the SQCC cohort showed a trend of positive correlation with BI (Fig. 2b). In contrast, a weak positive and negative correlation trend in the smoker group of the ADC cohort was observed (Fig. 2a).

**Fig. 3:**
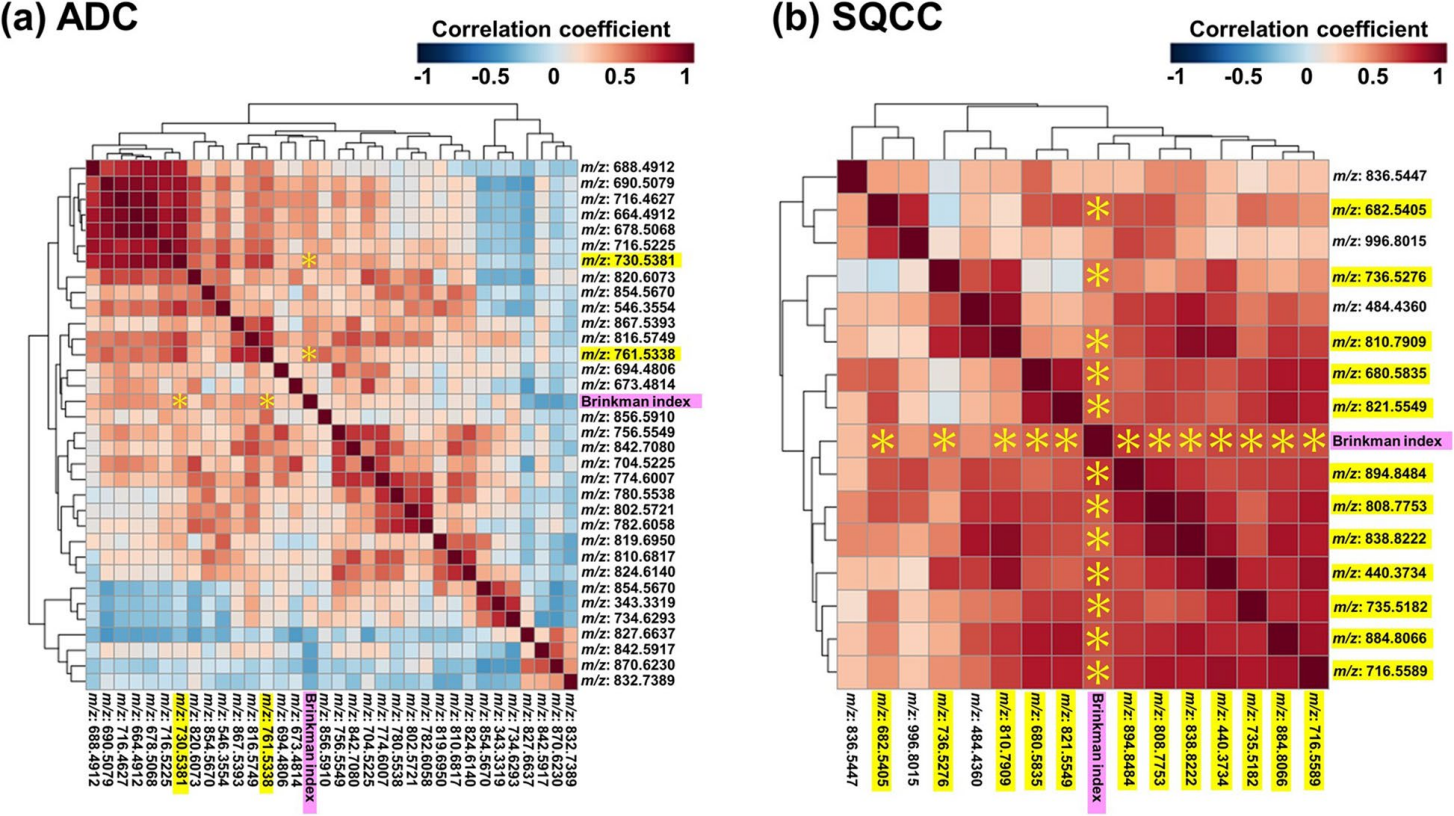
Correlation analysis between the screened lipid peaks and Brinkman index (BI). Two lipid peaks in the ADC cohort showed significant correlations with BI (**a**), while 12 lipid peaks showed significance in the SQCC cohort (**b**) (indicated as yellow highlighted *m/z*). A significant correlation was defined as Pearson correlation coefficient ≥ 0.5 ∩ *P*-value < 0.05 (shown as yellow asterisks). Abbreviations: ADC, Adenocarcinoma; BI, Brinkman index; *m/z*, Mass-to-charge ratio; SQCC, Squamous cell carcinoma

These findings led us to perform a correlation analysis between the screened lipid peaks and BI. Only two of the 29 lipid peaks in the ADC cohort (Fig. 3a) showed a significant correlation with BI. On the other hand, 12 of the 15 lipid peaks showed significance in the SQCC cohort (Fig. 3b). The correlation tables with *P*-values are presented in Supplemental file 2, sheet 2. From these results, we considered these lipid peaks that showed significant correlations with BI as lipids that were influenced by cigarette smoking.

### Identification of lipids that reflect postoperative recurrence and histopathological prognostic factors

**Fig. 4:**
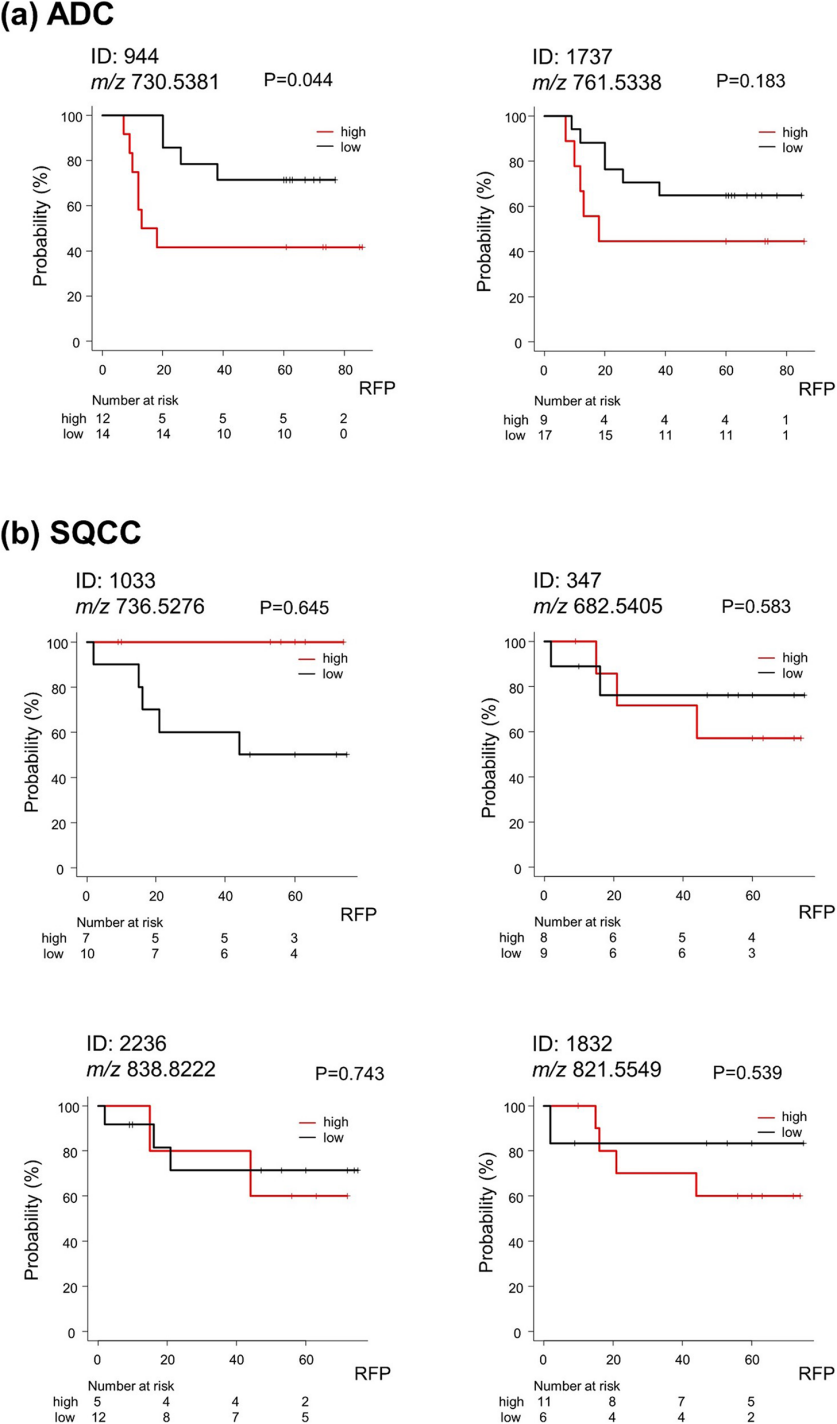
RFP curve analysis of lipid peaks with significant correlation with Brinkman index. **a** For *m/z* 730.5381 (ID: 944) in the ADC cohort, the high intensity group showed shorter RFP than the low intensity group. **b** For *m/z* 736.5276 (ID: 1033) in the SQCC cohort, recurrence was not seen in the high intensity group. Abbreviations: ADC, Adenocarcinoma; ID, Identification number; *m/z*, Mass-to-charge ratio; RFP, Recurrence-free period; SQCC, Squamous cell carcinoma

The fourteen lipid peaks showing a significant correlation with BI were subjected to AUC analysis to evaluate the discrimination ability between the recurrent and non-recurrent cases (Table 2). Two and four lipid peaks in the ADC and SQCC cohorts that showed AUC ≥ 0.6 were selected, respectively. Then, we performed an RFP curve analysis on these lipid peaks using the cutoff values calculated in the AUC analysis. In the case of *m/z* 730.5381 (ID: 944) in the ADC cohort, the RFP of the high intensity group was significantly shorter than in the low intensity group (*P* = 0.044, median RFP [MRFP]: 15.5 vs 60.5 months) (Fig. 4a). Notably, with an *m/z* of 736.5276 (ID: 1033) in the SQCC cohort, recurrence was not seen in the high intensity group. Accordingly, the *P*-value of this RFP analysis did not meet the significance level (*P* = 0.645, MRFP: 45.5 vs 56.0 months) regardless of the observed difference in recurrent prognosis between the low and high intensity groups. Hence, we regarded *m/z* 736.5276 as a valid predictor for postoperative recurrence.

**Table 2:** The rank of AUC analysis discriminating the recurrent and non-recurrent cases

Rank	ID	Average observed mass (*m/z*)	Theoretical mass (*m/z*)	Ion formula	AUC (95% CI)*
ADC					
**1**	**944**	**730.5379**	**730.5381**	**C40 H77 O8 N1 P1**^**+**^	**0.655 (0.427—0.882)**
**2**	**1737**	**761.5338**	**761.5338**	**C41 H78 O10 N0 P1**^**−**^	**0.612 (0.374—0.850)**
SQCC					
**1**	**1033**	**736.5285**	**736.5276**	**C42 H75 O7 N1 P1**^**+**^	**0.733 (0.490—0.976)**
**2**	**347**	**682.5405**	**682.5405**	**C43 H72 O5 N1**^**+**^	**0.700 (0.419—0.981)**
**3**	**2236**	**838.8222**	**838.8222**	**C53 H108 O5 N1**^**+**^	**0.617 (0.333—0.901)**
**4**	**1832**	**821.5550**	**821.5549**	**C43 H82 O12 N0 P1**^**−**^	**0.617 (0.301—0.932)**
5	2277	894.8485	894.8484	C56 H112 O6 N1^+^	0.583 (0.167—0.999)
6	2222	808.7753	808.7753	C51 H102 O5 N1^+^	0.567 (0.220—0.913)
7	2299	884.8066	884.8066	C57 H106 O5 N1^+^	0.550 (0.230—0.870)
8	20	440.3734	440.3734	C26 H50 O4 N1^+^	0.533 (0.247—0.820)
9	2219	810.7910	810.7909	C51 H104 O5 N1^+^	0.533 (0.244—0.823)
10	948	716.5590	716.5589	C40 H79 O7 N1 P1^+^	0.500 (0.218—0.782)
11	126	680.5835	680.5835	C41 H78 O6 N1^−^	0.433 (0.046—0.821)
12	1722	735.5182	735.5182	C39 H76 O10 N0 P1^−^	0.417 (0.065—0.768)

*Abbreviations*: *ADC* Adenocarcinoma, *AUC* Area under the ROC curve, *CI* Confidential interval, *ID* Identical number, *m/z* Mass to charge ratio, *SQCC* Squamous cell carcinoma

^*^Lipids with AUC ≥ 0.6 were selected as candidate lipid markers

The tandem mass spectrometry analysis of *m/z* 730.5381 and *m/z* 736.5276 in the ADC and SQCC cohorts were attempted using tandem mass spectral data. The mass spectrum peak of *m/z* 730.5381 was composed of three different PC isomers of PC (14:0_18:2), PC (16:1_16:1), and PC (16:0_16:2) (Additional file 1, Supplemental Fig. 2). We defined the combined area value of these three PC species as ‘PC cluster’. In contrast, the structural identification of *m/z* 736.5276 was not achieved because of its weak intensities in all samples. Using LipidSearch™ software, PC (O-14:1_20:5) was assigned as a candidate molecule of *m/z* 736.5276.

**Fig. 5:**
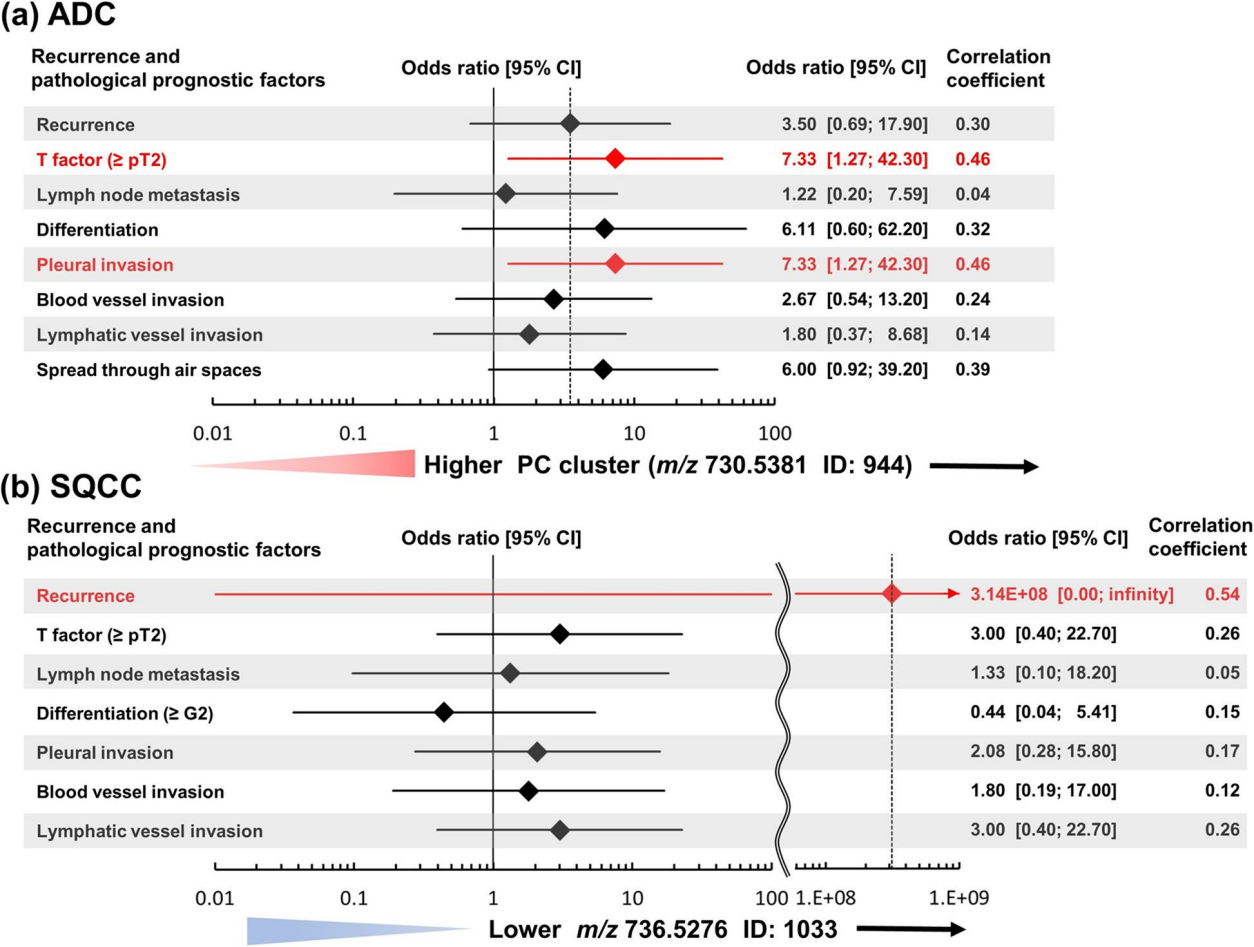
The higher intensity of the PC cluster and lower intensity of *m/z* 736.5276 reflect the likelihood of postoperative recurrence and histopathological prognostic factors. **a** The higher PC cluster level of the ADC cohort was significantly associated with greater likelihoods of progressed T-factor (≥ pT2) and pleural invasion (indicated in red). Recurrence, differentiation (≥ G2), and STAS showed tendencies of moderate likelihood, though they did not reach significance. **b** The lower *m/z* 736.5276 level of the SQCC cohort was strongly associated only with a greater likelihood of recurrence (indicated in red). Abbreviations: ADC, Adenocarcinoma; CI, confidence interval; ID, Identification number; *m/z*, Mass-to-charge ratio; PC, Phosphatidylcholine; SQCC, Squamous cell carcinoma; STAS, spread through air space

Then, we evaluated correlations between the identified lipids and recurrence or pathological prognostic factors with ORs. The higher PC cluster (*m/z* 730.5381) level of the ADC cohort was significantly associated with greater likelihoods of progressed T-factor (≥ pT2) (OR: 7.33, 95% confidence interval [CI]: 1.27–42.30, rs: 0.46) and pleural invasion (OR: 7.33, 95% CI: 1.27–42.30, rs: 0.46). Additionally, recurrence (OR: 3.50, 95% CI: 0.69–17.90, rs: 0.30), differentiation (≥ G2) (OR: 6.11, 95% CI: 0.60–62.20, rs: 0.32), and STAS (OR: 6.00, 95% CI: 0.92–39.20, rs: 0.39) showed tendencies of moderate likelihoods, though they did not reach statistical significance (Fig. 5a). The lower *m/z* 736.5276 level of the SQCC cohort was strongly associated only with a greater likelihood of recurrence (OR: 3.14E + 08, 95% CI: 0.00-infinity, rs: 0.54) (Fig. 5b).

With this result, we identified lipid biomarkers that reflected postoperative recurrence risk and pathological prognostic factors of lung ADC and SQCC patients who are smokers.

## Discussion

In this study, we identified lipid biomarkers influenced by cigarette smoking and reflected postoperative recurrence risk of lung ADC and SQCC. Overall, the findings are compatible with our initial hypothesis.

All the lipid peaks significantly influenced by cigarette smoking showed a positive correlation with BI; lipid peaks showing significant negative correlations were not detected. Previous studies have demonstrated negative correlations between cigarette smoking and lipid levels, mainly PC, in normal lung tissues. The mechanism of this negative correlation has largely been explained by decreased PC production in the alveolar type II cells [23–25]. The positive correlation we observed in this study between cigarette smoking and lipid levels in NSCLC tissues, the mechanism of which remains unknown, showed an inverse trend compared with that reported in normal lung tissues in previous studies. This mechanism may be explained by studies investigating the correlation between lipid metabolism and oxidative stress. Cigarette smoking generates reactive oxygen species (ROS) from free radicals, which is a significant oxidative stress factor for cells [29, 30]. Cancer cells exposed to oxidative stress produce a large number of lipid droplets that promotes stress resistance and homeostasis [31]. Actually, a higher intracellular ROS level is demonstrated to be associated with a higher lipid accumulation [32]. ROS damage mitochondria resulting in a further increase in ROS levels. Then, the increased ROS levels activate the sterol regulatory element binding protein pathway that leads to lipid accumulation [33].

A greater number of lipid peaks with significant positive correlations were identified in the SQCC cohort (12 peaks) than in the ADC cohort (two peaks). Cigarette smoking has the strongest association with the oncogenesis of SQCC and small cell carcinoma among all the lung cancer histological subtypes. The relative risk of non-ADCs associated with cigarette smoking is approximately ten while doubling the risk of ADC [28]. During cancer development, lipid metabolism is reprogrammed to satisfy the strong lipid avidity of the transformed cells. Endogenous lipid synthesis and uptake of exogenous lipids are enhanced to maintain a highly proliferative phenotype [13, 14]. Therefore, a greater number of lipids with a significant positive correlation with BI in the SQCC cohort may be attributable to an indirect causal relationship between cigarette smoking and lipid metabolism alteration mediated by oncogenesis.

Among the lipid peaks that showed a significant positive correlation with BI, the high intensity of one lipid peak (*m/z* 730.5381) in the ADC cohort was significantly associated with shorter RFP. The PC cluster (*m/z* 730.5381) in the ADC cohort was revealed to consist of three PC isomers, PC (14:0_18:2), PC (16:1_16:1), and PC (16:0_16:2), and the high-intensity level of this PC cluster was significantly associated with progressed T-factor (≥ pT2) and pleural invasion. According to the 8^th^ edition of the TNM classification for lung cancer, the pathological T-factor is determined by invasion diameter [4]. However, the reproducibility of invasion diameter measurements and the diagnostic concordance among pathologists are low, partially because of their subjective judgement [11, 34, 35]. The routine evaluation method for pleural invasion also varies in pathologists [36]; this may hinder objective validation. In contrast, the PC cluster identified in our study reflected the likelihoods, displayed as ORs, of these two pathological prognostic factors; these ORs were considered more objective than conventional pathological evaluation. If the prognostic value of the PC cluster is validated in other cohorts, these lipids could be developed as an alternative parameter for the conventional pathological prognostic factors. Although the higher PC cluster showed tendencies of a moderate likelihood for recurrence, differentiation (≥ G2), and STAS, the ORs were not statistically significant; this may be attributable to the small sample size of the cohort.

The species in the PC cluster included 16-carbon fatty acids (FA) of FA (16:0) (palmitic acid) and FA (16:1) (palmitoleic acid). De novo synthesis of FA (16:0) is enhanced by increased expression of fatty acid synthase (FASN) in various cancers [14]. FA (16:0) is desaturated by stearoyl-CoA desaturases (SCDs) to form FA (16:1) [14]. Notably, PC species that contain FA (16:1) are increased in some cancer tissues compared with normal tissues, including lung ADC [37, 38]. Because FASN and SCDs that produce these saturated and unsaturated FAs are suggested to play essential roles in cancer progression [14, 39, 40], our findings that higher PC species, including FA (16:0) and FA (16:1), reflected shorter RFP and disease progression are compatible with these previous reports.

The lower intensity of one lipid peak (*m/z* 736.5276) in the SQCC cohort was strongly associated with shorter RFP and postoperative recurrence. Nevertheless, associations between *m/z* 736.5276 and pathological prognostic factors remained weak. Therefore, *m/z* 736.5276 may reflect postoperative recurrence risk in which conventional pathological prognostic factors cannot be explanatory variables. The LipidSearch™ software suggested that the candidate molecule for *m/z* 736.5276 is PC (O-14:1_20:5). To the best of our knowledge, the bioactivity of PC (O-14:1_20:5) in cancer biology is unknown. We believe that *m/z* 736.5276 can be developed as an objective predictor for postoperative recurrence.

Lastly, our previous studies reported SM (d35:1) and SM (t34:1) in NSCLC as candidate predictors for postoperative recurrence after radical surgery [21, 22]. However, we did not identify SM species that reflect BI and postoperative recurrence risk in this study. The volcano plots described on lipid peaks for which SM species were assigned as candidate molecules by LipidSearch™ software showed only one significant lipid peak (*m/z* 819.6950 [ID: 2132]) in the smoker group of the ADC cohort; the assigned candidate molecule was SM (t41:0) (Additional file 1, Supplemental Fig. 3). This lipid peak did not show significant correlation with BI in the subsequent correlation analysis (Fig. 3a). The candidate lipid biomarkers identified in this study were considered distinctive from the two SM species we have reported previously in that these lipids can reflect both BI and recurrence risk.

Several limitations in this study must be acknowledged. First, our study was conducted with a retrospective design and a small sample size. Therefore, reproducibility could not be validated using other cohorts. On screening of the *m/z* 730.5381 (ID: 944) and *m/z* 736.5276 (ID: 1033) using t-test in the volcano plots, the post hoc powers provided by sample sizes of ADC and SQCC cohorts were low as 39.8% and 56.8%, respectively: the low post hoc powers were attributable to the small sample size. Based on standard deviations of *m/z* 730.5381 (ID: 944) and *m/z* 736.5276 (ID: 1033) observed in this study, the required sample sizes for a detection power of 80% with a two-sided significance level of *P* < 0.05 were 30 and 16 patients per group for ADC and SQCC cohorts. A future large cohort validation study is necessary to develop the lipids identified in this study into robust prognostic factors. Second, the MS peak of the PC cluster composed of three different PC isomers could not be isolated into respective isomers because their RTs were too close to one another. Therefore, we could not calculate the area value of single PC isomers, and evaluating the prognostic impact of single PC isomers was difficult. The difference in biological activities among the three PC species is currently unknown. Accordingly, there is no obvious necessity to isolate these PC isomers to evaluate the prognostic impact. Third, structural identification of *m/z* 736.5276 could not be achieved because of its weak intensity. Hence, it remained a candidate molecule with database exploration. The weak intensity may be attributed to the small amount of extracted lipid samples. Further experiments using specimens from another cohort are needed for molecule identification. Fourth, in the screening of the lipid peaks associated with postoperative recurrence using the KM method for RFP analysis, 10 (38%) patients in the ADC cohort and one (6%) patient in the SQC cohort received adjuvant chemotherapy that was previously shown to reduce the postoperative recurrence risk [41, 42]. Accordingly, the bias of the adjuvant chemotherapy could not be eliminated from the RFP analysis. Fifth, In the ADC cohort, more female patients were enrolled in the never smoker group (75%) than in the smoker group (5.9%); thereby, the difference demonstrated in the volcano plot of the ADC cohort may have been biased by the sex difference. However, we performed further correlation analysis for screening lipids showing a significant correlation with BI. Therefore, we regarded the screened lipids as influenced by cigarette smoking. Further validation study using another cohort with no background difference is expected. Sixth, LC–MS/MS is not standard in routine clinical examinations. However, LC–MS/MS as an optional diagnostic modality in cancer screening has advanced recently [43, 44]. Introducing LC–MS/MS for screening patients with a high risk of postoperative recurrence is strongly expected.

## Conclusions

From our data, we propose three PC isomers, PC (14:0_18:2), PC (16:1_16:1), and PC (16:0_16:2), and a lipid peak of *m/z* 736.5276 as novel candidate biomarkers for postoperative recurrence risk in lung ADC and SQCC patients that smoke. Our findings may contribute to developing an objective method for evaluating postoperative recurrence risk and a qualified postoperative therapeutic strategy for smoking NSCLC patients.

## Supplementary Information

**Additional file 1.**

**Additional file 2.**

## Data Availability

The dataset supporting the conclusions of this article is included within the Additional Files.

## Abbreviations

ADC: Adenocarcinoma
AUC: Area under the ROC curve
BI: Brinkman index
CEA: Carcinoembryonic antigen
CI: Confidence interval
CT: Computed tomography
CYFRA: Cytokeratin 19 fragment
LC–MS/MS: Liquid chromatography-tandem mass spectrometry
*m/z*: Mass-to-charge ratio
MRPF: Median recurrence-free period
NSCLC: Non-small cell lung cancer
PC: Phosphatidylcholine
RFP: Recurrence-free period
ROC: Receiver operating characteristic
ROS: Reactive oxygen species
rs: Spearman’s rank correlation coefficient
RT: Retention time
SCC: Squamous cell carcinoma antigen
SM: Sphingomyelin
SQCC: Squamous cell carcinoma
STAS: Spread through air space
TNM: Tumor, lymph node, and metastasis
TTF-1: Thyroid transcription factor-1
