# Supplementary material for: Lipid biomarkers that reflect postoperative recurrence risk in lung cancer patients who smoke: a case–control study
Source: Lipids Health Dis. 2023 Jan 28;22:15. doi: 10.1186/s12944-023-01778-3 (PMC9883920; doi:10.1186/s12944-023-01778-3)
Supplement: Supplementary file 1 — Additional file 1. [file 12944_2023_1778_MOESM1_ESM.pdf]

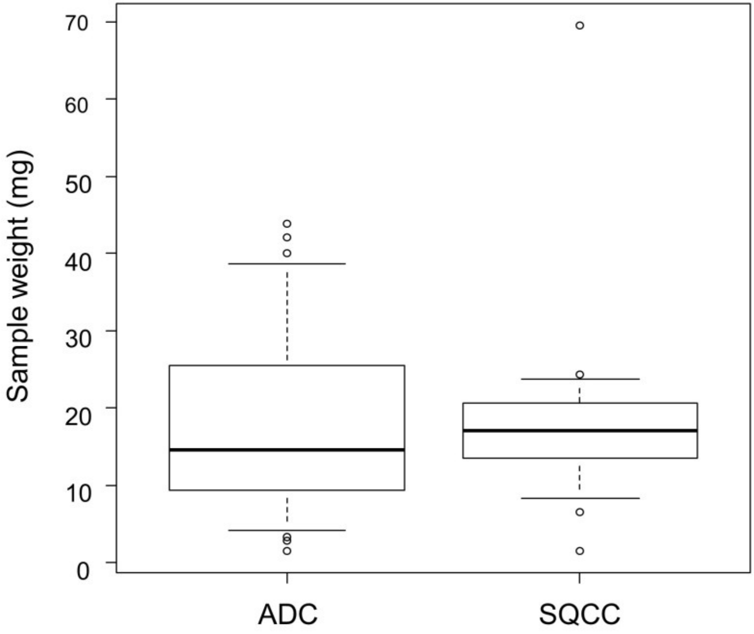

Supplemental Figure 1. Frozen tissue sample weights. The average sample weights recruited in this study were 18.2 mg (range: 1.5–43.9 mg) and 18.6 mg (range: 1.5–69.6 mg) in the ADC and SQCC cohorts, respectively. Abbreviations: ADC, Adenocarcinoma; SQCC, Squamous cell carcinoma.

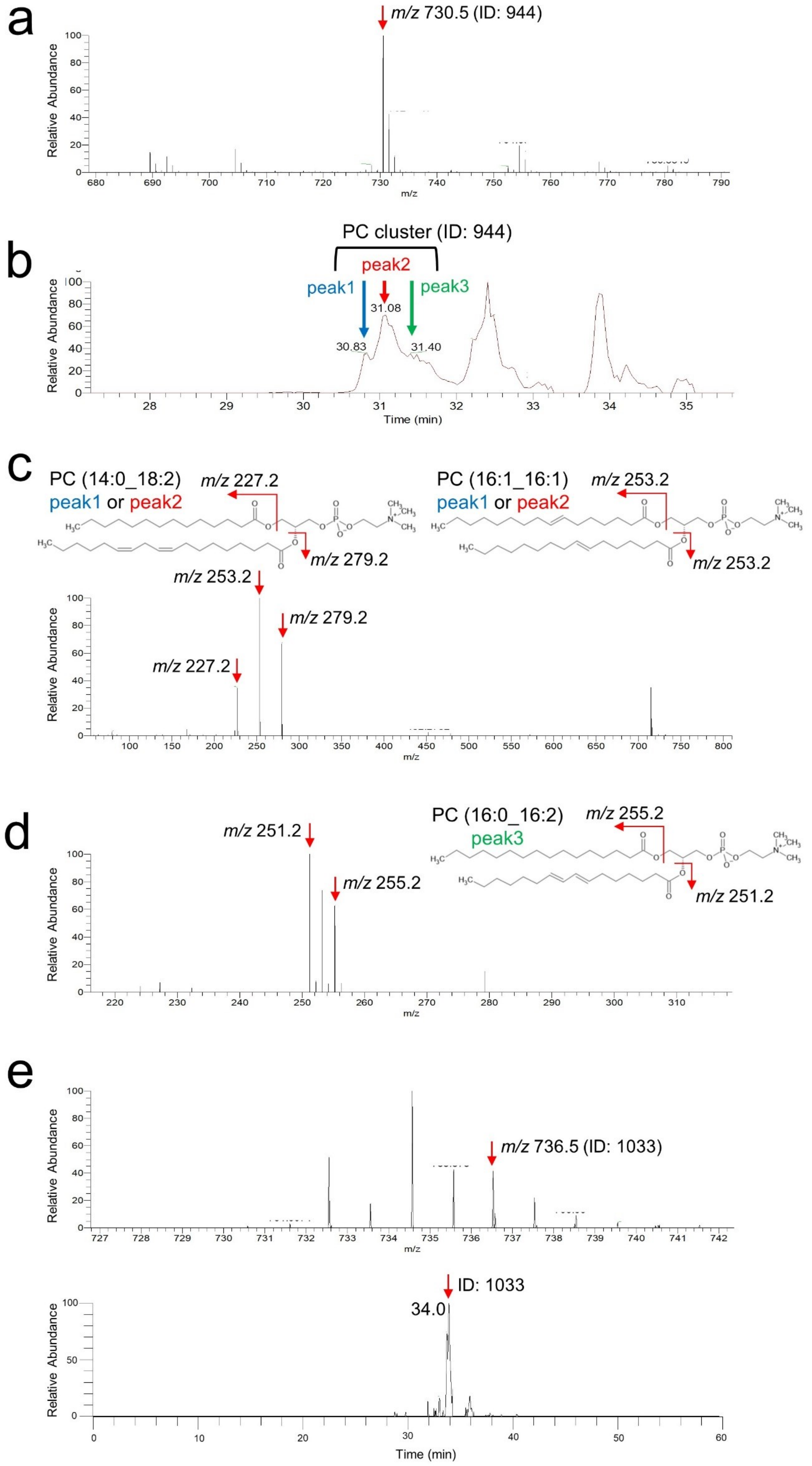

Supplemental Figure 2. The MS findings and MS/MS analysis of the identified lipids.
The MS peak of *m/z* 730.5 (ID: 944) (a) identified in the adenocarcinoma cohort was regarded as a PC cluster composed of three isomers in the chromatogram (peak 1–3) (b). (c) Among MS/MS analysis of the three isomers, peaks 1 and 2 were compatible as PC (14:0_18:2) or PC (16:1_16:1), demonstrating fragments of FA (14:0) (*m/z* 227.2), FA (18:2) (*m/z* 279.2), and FA (16:1) (*m/z* 253.2). (d) For peak 3, the detected fragments, FA (16:0) (*m/z* 255.2) and FA (16:2) (*m/z* 251.2), were compatible as product ions from PC (16:0_16:2). (e) For the MS peak of *m/z* 736.5 (ID: 1033) (upper panel) in the squamous cell carcinoma cohort, the corresponding peak in the chromatogram had a single peak (lower panel). However, the MS/MS analysis was not performed because of its weak signal intensity.
Abbreviations: FA, Fatty acid; ID, Identification number; MS, Mass spectrometry; MS/MS, Tandem mass spectrometry; PC, Phosphatidylcholine.

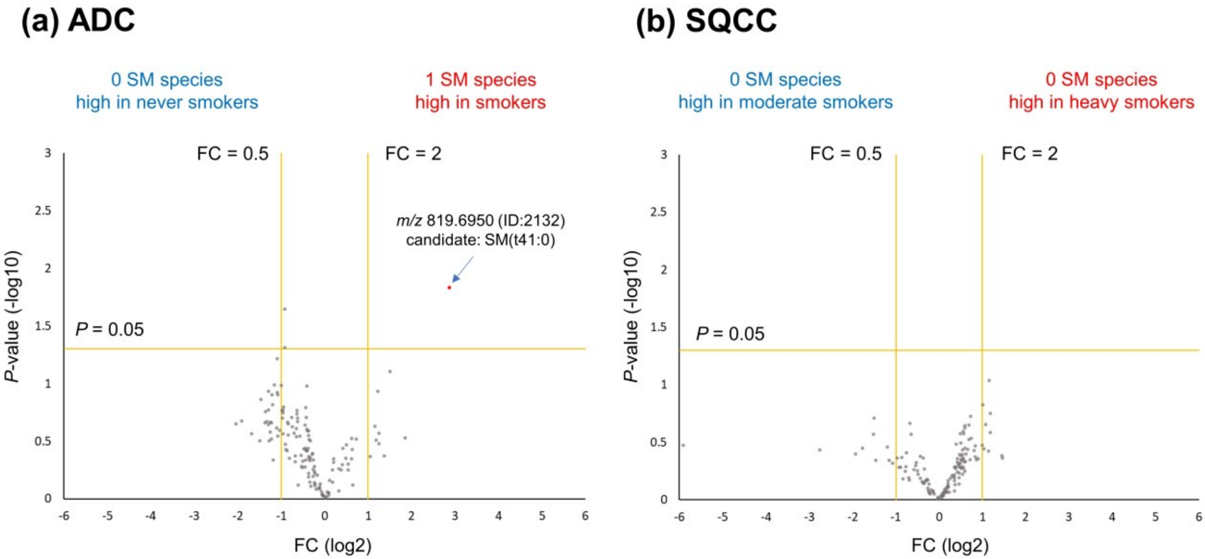

Supplemental Figure 3. Volcano plots described on lipid peaks for which SM species were assigned as candidate molecules by LipidSearch™ software.
Only one significant lipid peak (*m/z* 819.6950 [ID: 2132]) in the smoker group of the ADC cohort was identified; the assigned candidate molecule was SM (t41:0) (a). On the other hand, no significant lipid peak was identified in the SQCC cohort (b).
Abbreviations: ADC, Adenocarcinoma; FC, Fold change; SM, sphingomyelin; SQCC, Squamous cell carcinoma.
